# Exercise and physiological monitoring in isolated, confined and extreme environments and space analogues: A scoping review

**DOI:** 10.1113/EP093836

**Published:** 2026-11-01

**Authors:** Gustavo S. O. Lima, Kaline V. F. Dinis, Leonardo P. Silva, Nayanne B. Sousa, Maria Keiliane F. S. Silva, Danilo C. Talhari, Josélia S. Almeida, Michele M. Moraes, Rosa M. E. Arantes, Marcos A. Nascimento, Thiago T. Mendes

**Affiliations:** ^1^ Graduate Program in Medicine and Health (PPGMS) Federal University of Bahia (UFBA) Salvador Bahia Brazil; ^2^ Department of Chemical Engineering Federal University of Bahia (UFBA) Salvador Bahia Brazil; ^3^ Department of Physical Education State University of Maranhão (UEMA) São João dos Patos Brazil; ^4^ Department of Applied Physiology and Kinesiology University of Florida Gainesville Florida USA; ^5^ Institute of Biological Sciences Universidade Federal de Minas Gerais (UFMG) Belo Horizonte Minas Gerais Brazil

**Keywords:** Antarctica, exercise countermeasures, heart rate variability, sleep, space analogue

## Abstract

This scoping review synthesized evidence on physical activity/exercise and physiological monitoring in isolated, confined and extreme (ICE) environments, with emphasis on Antarctic missions and space analogues. ICE exposures combine cold stress, confinement, disrupted light–dark cycles and psychosocial strain, which can challenge musculoskeletal integrity, cardiovascular regulation, sleep/circadian function and cognition. Following Joanna Briggs Institute (JBI) methodology and PRISMA‐ScR guidance, we searched Web of Science, Embase, Scopus and PubMed/Medline, complemented by grey literature and AI‐assisted citation chasing. Eligible reports included human studies in ICE/analogue settings that implemented or quantified physical activity/exercise and/or physiological monitoring. Seventeen studies published between 2003 and 2025 met inclusion criteria and were mapped across ICE characteristics, exercise protocols, monitoring technologies, outcomes and research gaps. Exercise was reported as a potential countermeasure to attenuate selected aspects of deconditioning and support sleep and psychophysiological adaptation, particularly in bed rest and simulated confinement studies. Antarctic field studies more often characterized spontaneous physical activity, sleep/activity rhythms and field‐adapted monitoring. Assessment strategies varied, ranging from self‐reported logs and actigraphy in operational field settings to laboratory‐grade measures in bed rest and simulated confinement studies. Overall, exercise emerged as a pragmatic, domain‐specific countermeasure against deconditioning in ICE settings, with effectiveness varying by modality and physiological target. However, heterogeneity in exposure characterization, exercise dose and monitoring methods, despite feasible wearable‐based capture of behavioural and physiological signals, limited comparability and mechanistic inference. Future ICE research should prioritize harmonized reporting, standardized monitoring and longitudinal designs linking exercise exposure to physiological, behavioural and operational outcomes.

## Abstract (highlights)

- **What is the topic of this review?**
  This review synthesizes evidence on activity/exercise and physiological monitoring in isolated, confined and extreme environments, focusing on Antarctic stations, confinement and microgravity analogues.
- **What advances does it highlight?**
  Evidence from 17 studies published between 2003 and 2025 suggests that exercise, including flywheel training and artificial gravity in bed rest and microgravity analogues, preserved musculoskeletal and cardiovascular outcomes. In confinement, exercise showed cognitive and psychophysiological benefits, varying by design, exposure and monitoring. Antarctic studies described activity, sleep/activity patterns and winter reductions. Heterogeneity limited comparability, supporting reporting on sleep, heart rate variability and exercise workload.

## 1 INTRODUCTION

Human exposure to isolated, confined and extreme (ICE) environments provides a unique model to study integrative homeostatic and adaptive physiology under combined stressors. Antarctic missions and space analogues impose constraints that include cold exposure, confinement, altered light–dark cycles and psychosocial strain, which can disrupt regulatory systems and challenge operational performance (Zivi et al., 2020). The physical and environmental features of these settings, including latitude‐dependent photoperiod, low ambient temperature and altitude, are consistently associated with disturbances in sleep and circadian regulation. These features can interact dynamically with operational workload and individual physiological state to shape multisystem adaptive responses (Collet et al., 2015; Steinach et al., 2016). Across ICE and analogue contexts, reported outcomes are multisystemic. Confinement or unloading protocols are linked to musculoskeletal deconditioning and bone–muscle loss, highlighting the central role of mechanical loading as a determinant of adaptive capacity (Gabel et al., 2022; Moraes et al., 2023; Roalf et al., 2025).

Autonomic cardiovascular regulation is often discussed as an integrative marker of adaptation and recovery status in extreme environments, although interpretation is sensitive to measurement context and concurrent exposures (Lima et al., 2024; Moraes et al., 2018). Additional domains relevant to ICE physiology include thermoregulatory adaptation during fieldwork and changes in inflammatory/metabolic profiles during combined environmental and behavioural constraints (Goto, 2025; Moraes et al., 2023).

Exercise and structured physical activity are widely used as countermeasures in space analogues and operational missions because they can attenuate deconditioning and support autonomic cardiovascular regulation, musculoskeletal function and neurocognitive performance. Controlled confinement and analogue studies have reported beneficial effects of exercise on physiological deconditioning and selected neurocognitive outcomes, although responses vary with exercise dose, timing and exposure characteristics (Kraft et al., 2003; Schneider et al., 2013).

The evidence remains scattered across heterogeneous designs, exposure contexts and monitoring approaches. To support integrative interpretation, it is useful to conceptualize ICE research within an exposure‐to‐response framework, in which environmental and operational stressors act through mediators such as circadian and sleep regulation, autonomic cardiovascular control, neuroendocrine stress responses, thermoregulation, inflammatory signalling and deconditioning pathways, shaping multisystem outcomes and performance (Collet et al., 2015; Moraes et al., 2022). Given this scenario, the aim of the present study was to map and synthesize the available scientific literature on strategies for physical activity assessment and monitoring in ICE environments and their analogues. The practical question was: what evidence is available on physical activity and physiological monitoring in ICE environments and their analogues?

## 2 MATERIALS AND METHODS

### 2.1 Ethics statement

This study is a scoping review of published and publicly available reports. No new data were collected from human participants or animals; therefore, institutional ethics approval and informed consent were not required.

### 2.2 Search strategy

The proposed scoping review was conducted according to the Joanna Briggs Institute (JBI) methodology for evidence synthesis and scoping reviews. In addition, it followed the Preferred Reporting Items for Systematic Reviews and Meta‐Analyses extension for Scoping Reviews (PRISMA‐ScR), ensuring transparency and reproducibility in the processes of searching, selecting and analysing the evidence (Munn et al., 2018). This approach was selected because it allows mapping the volume, characteristics and scope of the available literature, providing a comprehensive overview of the topic and supporting the identification of evidence gaps and research priorities (Jordan & Pilla, 2024).

A scoping review design was chosen because of the heterogeneity and relative scarcity of study designs, outcomes and monitoring methods reported in research on physical activity and physiological monitoring in ICE environments. Searches were conducted in May 2026. A comprehensive and structured search was performed in major bibliographic databases relevant to the topic (Web of Science, Embase, Scopus and PubMed/Medline), using combinations of controlled vocabulary terms from Medical Subject Headings (MeSH) and Health Sciences Descriptors (DeCS), in addition to free‐text keywords, linked by Boolean operators (‘AND’ and ‘OR’).

The research strategy was structured into three components: (1) environment and exposure context, including Antarctic/polar environments, space missions and simulations, terrestrial microgravity analogues, hypobaric/high‐altitude conditions, and underwater or subaquatic habitats; (2) isolation and confinement, including prolonged confinement, hibernation, quarantine, airtight isolation and long‐duration missions; and (3) exercise, physical activity and physiological monitoring, including training modalities, countermeasure strategies, wearable devices/activity monitoring, and outcomes related to deconditioning.

The search combined terms for ICE/analogue environments, isolation or confinement, and exercise, physical activity or physiological monitoring. Database‐specific strategies are provided in the Appendix Data A1.

In addition to database searching, grey literature was consulted through the Brazilian Digital Library of Theses and Dissertations (BDTD). An artificial intelligence‐assisted citation‐chasing (‘snowball’) strategy was also conducted using Research Rabbit APP (https://researchrabbitapp.com/) to identify additional relevant studies from reference lists and citation networks.

### 2.3 Inclusion and exclusion criteria

Following the JBI PCC (population, concept, context) framework, we included studies investigating adults (≥18 years of age) exposed to ICE conditions or analogous environments. Regarding the concept, eligible studies reported physical activity or structured exercise (supervised or unsupervised) and/or physiological or psychophysiological monitoring strategies, physical activity and exercise were treated as distinct constructs, with exercise defined as planned, structured and repetitive physical activity aimed at maintaining or improving physical fitness.

Eligible contexts included Antarctic stations and field camps, long‐duration confinement protocols, space‐analogue simulations and terrestrial microgravity models, including head‐down‐tilt bed rest and dry immersion, when explicitly framed as spaceflight or operational extreme‐environment analogues. Studies using immobilization exclusively as clinical or rehabilitation models, studies unrelated to physical activity/exercise or physiological monitoring, and studies conducted in conventional environments without isolation, confinement or environmental extremes were excluded.

### 2.4 Types of design

Experimental and quasi‐experimental study designs were considered for inclusion, including randomized and non‐randomized clinical trials. Analytical observational studies (prospective and retrospective cohorts, case–control studies and analytical cross‐sectional studies) were also eligible. Descriptive observational designs (case series, case reports and descriptive cross‐sectional studies) were included, in addition to grey literature sources reporting original data, such as theses/dissertations and institutional/technical research reports. This broader inclusion strategy was adopted because research in ICE environments, particularly missions to Antarctica and space analogues, is characterized by small sample sizes, operational and ethical constraints, and methodological limitations inherent to field‐ and mission‐based investigations, which often reduce the feasibility of large controlled studies.

### 2.5 Study selection/source of evidence

Following the searches, 1467 records were identified through database and register searches. In addition, citation searching identified 11 additional records, resulting in 1478 records/reports identified overall. The 1467 records retrieved from database and register searches were exported to EndNote (Clarivate Analytics, PA, USA), where duplicate records (*n* = 882) were removed. The remaining unique records (*n* = 585) were transferred to a customized Excel spreadsheet and subsequently uploaded to Rayyan (https://new.rayyan.ai/) to support screening and data extraction.

In total, 585 records were screened against predefined inclusion and exclusion criteria. After title and abstract screening, 557 records were excluded for not meeting eligibility criteria. Twenty‐eight reports were sought for full‐text retrieval; however, nine could not be obtained because the full text was unavailable or inaccessible despite retrieval attempts. Therefore, 19 reports were assessed in full text for eligibility.

After full‐text assessment, five reports were excluded because they did not report eligible physical activity/exercise exposure or predefined physiological or psychophysiological monitoring outcomes aligned with the review question. Thus, 14 studies were included from database and register searching. In addition, three studies were identified and included through citation searching, resulting in 17 studies included in the final qualitative synthesis. The study selection process is presented in the PRISMA‐ScR flow diagram (Figure 1).

**FIGURE 1:**
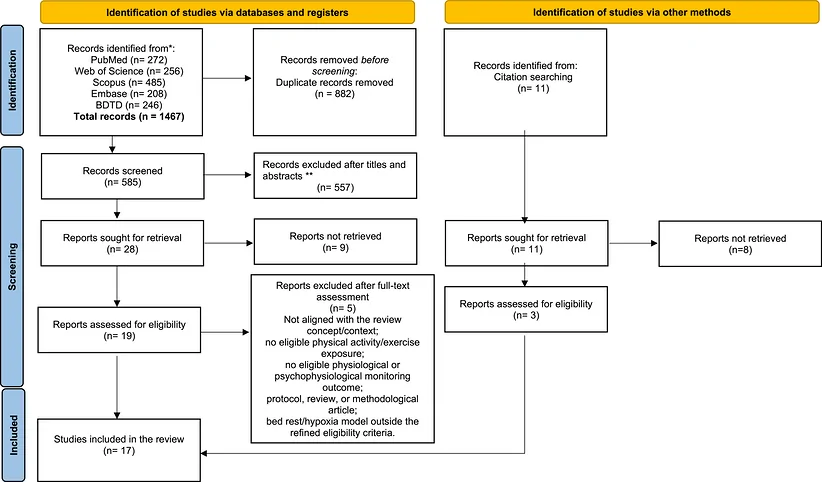
PRISMA‐ScR flow diagram of study identification, screening, eligibility assessment and inclusion. Source: Page MJ, et al. BMJ 2021;372:n71. https://doi.org/10.1136/bmj.n71. This work is licensed under CC BY 4.0. To view a copy of this license, visit https://creativecommons.org/licenses/by/4.0/

### 2.6 Data extraction and data analysis

Data extracted from the 17 reports included were collected independently by two reviewers using a standardized extraction form and organized using the following descriptive variables: author/year, study design, setting, intervention or exposure, monitored variables, technology used, main outcomes and limitations. This structure enabled comparison across designs and contexts, in addition to identification of patterns and gaps in the available evidence.

Discrepancies in the completion of the extraction matrix were discussed and resolved by consensus, with adjudication by a third reviewer when necessary, ensuring consistency throughout the process.

The analysis took place in two stages. First, studies were characterized by intervention or monitoring approach, including structured exercise, spontaneous physical activity and monitoring strategies; exposure environment, including Antarctica, space analogues, head‐down‐tilt bed rest as a spaceflight analogue, and prolonged confinement; and assessed domains, including sleep, body composition, cognition, musculoskeletal integrity and cardiovascular function. Second, findings were synthesized narratively and summarized in Table 1, allowing visualization of outcome patterns, monitoring technologies used and the main methodological limitations reported across studies (Munn et al., 2018). This inductive approach supported mapping of the current evidence on physical activity and physiological monitoring in ICE environments and identified gaps to inform future research.

**TABLE 1:** General characteristics of the studies included in the review (*n* = 17).

Author/year	Type of study	Environment	Intervention/exposure	Related variables
(1) Alkner & Tesch (2004)	Experimental/intervention (resistance exercise trial)	Space simulation chamber confinement (110 days)	Strength training with a gravity‐independent system	Strength, lean mass and dynamic performance (work and power)
(2) Schneider et al. (2010)	Experimental/intervention (exercise) research report	Confinement simulation MARS105 (105 days)	Incremental exercise bike test (15 W +15 W/min up to 85% of maximum load)	Cardiovascular, physiological and psychological
(3) Schneider et al. (2013)	Experimental/intervention (exercise) research report	Spaceflight simulation confinement (MARS500) (520 days)	Structured physical exercise during confinement (passive running on a treadmill, stationary cycling and resistance training)	Prefrontal cortex activity, cognition and psychophysiological responses
(4) Kraft et al. (2003)	Observational/experimental (longitudinal)	Confinement/simulation in an isolated environment (110 and 240 days)	Measurement of perceived exercise load (modified RPE) + reports of motivation and body weight	Exercise load index; body mass
(5) Roalf et al. (2025)	Observational/experimental (longitudinal)	Concordia Station Isolation (Antarctica), 12 months	Exposure to Antarctic confinement with measures of exercise, sleep and cognitive performance	Neuroimaging: grey matter volume, CSF, ventricles, sleep, cognition and psychological variables
(6) Rittweger et al. (2005)	Controlled clinical trial	Terrestrial simulation of microgravity: confinement in an anti‐orthostatic bed inclined at −6° for 90 days	Resistance exercise on flywheel + Pamidronate (AREDIA^®^)	Muscle mass, bone density and strength
(7) Collet et al. (2015)	Observational/experimental (longitudinal)	Isolation of Concordia and Dumont d'Urville stations (Antarctica), 12 months	Natural exposure in an ICE environment, monitoring spontaneous routine	Sleep; nighttime energy expenditure; daytime activity
(8) Tellez et al. (2016)	Mixed observational quasi‐experimental (two studies: normobaric confinement in hypoxia and hypobaric confinement)	Isolation at station (Concordia), 13 months	2×/day moderate cycling (1 h; ∼50% peak power hypoxia); control = no exercise; usual physical activity, self‐reported duration/intensity	Sleep; physical activity; body composition
(9) Steinach et al. (2016)	Observational/experimental (longitudinal)	Isolation station in Antarctica (Neumayer II and III) (seven wintering campaigns, 2008–2014, duration 13 months each)	Natural exposure in an ICE environment, monitoring spontaneous routine	Sleep; physical activity; local solar radiation
(10) Belavý et al. (2011)	Experimental (simulated test)	Terrestrial simulation of microgravity: confinement in an anti‐orthostatic bed at −6° for 90 days	Spinal mobilization exercises + flywheel	Lumbar function, paravertebral muscles, spinal health
(11) Moraes et al. (2018)	Experimental/intervention study (longitudinal)	Camping in Antarctica (24 days)	Field exercise in cold environments; continuous recording of HR, temperature and distance; pre/post ${\dot{V}}_{O_{2}\max}$ tests	${\dot{V}}_{O_{2}\max}$ (incremental), HR, HRV, skin and core temperature, lactate, perceived exertion and thermal acclimatization
(12) Garcia (2024)	Observational/experimental (longitudinal) (master's thesis)	Comandante Ferraz Antarctic Station, Brazil (244 days)	Monitoring sleep and physical activity during summer and winter (actigraphy)	Sleep duration and efficiency, naps, daily physical activity (M10AF), seasonality
(13) Smith et al. (2008)	Randomized/controlled bed rest countermeasure study	60 days of simulated microgravity in women; bed rest model	Supine treadmill exercise within lower‐body negative pressure combined with flywheel resistive exercise	Bone mineral density; bone resorption and formation markers; lean tissue/musculoskeletal outcomes; countermeasure efficacy
(14) Kramer et al. (2021)	Randomized controlled trial; artificial gravity countermeasure study	60 days of head‐down‐tilt bed rest; spaceflight analogue	Daily 30 min exposure to artificial gravity by short‐arm centrifugation; continuous or intermittent protocols	Aerobic exercise capacity; muscle function; knee‐extensor strength/power; neuromuscular deterioration; physical capacity
(15) Kramer et al. (2020)	Experimental/physiological countermeasure study	60 days of head‐down‐tilt bed rest; AGBRESA spaceflight analogue	Daily short‐arm centrifugation to elicit artificial gravity	Cardiorespiratory demand; neuromuscular demand; heart rate; oxygen uptake/energy demand; muscle activation or neuromuscular loading
(16) Hedge et al. (2025)	Experimental/intervention study; bed rest countermeasure trial	60 days of head‐down‐tilt bed rest; BRACE spaceflight analogue	Exercise during artificial gravity; combined exercise and artificial gravity countermeasure	Cardiorespiratory fitness; aerobic capacity; orthostatic tolerance; cardiovascular deconditioning
(17) Arbeille et al. (2024)	Experimental/intervention study; physiological monitoring and countermeasure trial	55 days of head‐down‐tilt bed rest	Exercise combined with artificial gravity and exercise‐only countermeasures	Organ morphology; blood vessel morphology; vascular/ultrasound measures; cardiovascular and organ adaptations

Abbreviations: AGBRESA, Artificial Gravity Bed Rest with the European Space Agency; HR, heart rate; HRV, heart rate variability; M10AF, actigraphy‐derived activity metric during the most active 10 h period; RPE, rating of perceived exertion; ${\dot{V}}_{O_{2}\max}$, maximum oxygen consumption.

## 3 RESULTS

### 3.1 Study selection

A total of 17 studies were included in qualitative synthesis. The study selection process is summarized in the PRISMA‐ScR flow diagram (Figure 1).

### 3.2 Study characteristics

Included studies were published between 2003 and 2025 and were conducted in ICE environments or in space‐analogue/simulated confinement settings. Three contextual categories were represented: (1) Antarctic station and field camp exposures (Collet et al., 2015; Garcia, 2024; Moraes et al., 2018; Roalf et al., 2025; Steinach et al., 2016; Tellez et al., 2016); (2) long‐duration simulated confinement and space‐analogue missions (Alkner & Tesch, 2004; Kraft et al., 2003; Schneider et al., 2010, 2013); and (3) head‐down‐tilt bed rest and artificial gravity protocols as spaceflight analogue models (Arbeille et al., 2024; Belavý et al., 2011; Hedge et al. 2025; Kramer et al., 2020, 2021; Rittweger et al., 2005; Smith et al., 2008).

Samples included military personnel, civilians and researchers participating in polar missions, in addition to volunteers enrolled in spaceflight analogues. Study designs ranged from randomized controlled trials and non‐randomized experimental interventions to observational longitudinal and mixed methods designs. Data collection periods ranged from 24 days (Moraes et al., 2018) to 520 days (Schneider et al., 2013), with bed rest protocols spanning 55–90 days (Arbeille et al., 2024; Hedge et al. 2025; Kramer et al., 2020, 2021; Smith et al., 2008) and Antarctic overwintering protocols lasting ≤13 months (Collet et al., 2015; Steinach et al., 2016). General characteristics of the included studies (environment, intervention/exposure and related variables) are summarized in Table 1.

### 3.3 Interventions/exposures and outcome domains

Across the 17 included studies, exercise and physical activity exposures varied by context, structure and delivery mechanism. Three exposure categories were identified: (1) structured exercise prescriptions, including resistance, flywheel, treadmill, cycling and combined protocols in simulated confinement and bed rest protocols (Alkner & Tesch, 2004; Belavý et al., 2011; Rittweger et al., 2005; Schneider et al., 2010, 2013; Smith et al., 2008); (2) artificial gravity, with or without exercise, delivered by short‐arm centrifugation during head‐down‐tilt bed rest (Arbeille et al., 2024; Hedge et al., 2025; Kramer et al., 2020, 2021); and (3) naturalistic physical activity monitoring in Antarctic ICE settings, including spontaneous routines and seasonal activity patterns (Collet et al., 2015; Garcia, 2024; Steinach et al., 2016). Additional Antarctic studies assessed structured aerobic exercise during normobaric hypoxia or field‐based exercise during camp exposure (Moraes et al., 2018; Tellez et al., 2016). Outcomes clustered around musculoskeletal and functional end points, cardiorespiratory and vascular responses, sleep and activity rhythms, and cognitive or psychophysiological measures.

### 3.4 Monitoring technologies and measurement approaches

Monitoring strategies ranged from laboratory‐grade systems to field‐adapted wearable solutions (Table A1). Laboratory‐based analogues used flywheel devices, short‐arm centrifugation, dual‐energy X‐ray absorptiometry (DXA), ultrasound, EEG and MRI to assess musculoskeletal, cardiovascular, vascular, neurophysiological and body composition outcomes (Arbeille et al., 2024; Kramer et al., 2020, 2021; Roalf et al., 2025; Schneider et al., 2010, 2013; Smith et al., 2008). Antarctic and field studies relied mainly on polysomnography, actigraphy/accelerometry, heart rate monitoring, thermal sensors, ergometry and perceived exertion to capture sleep, activity and physiological responses under operational constraints (Collet et al., 2015; Garcia, 2024; Moraes et al., 2018; Steinach et al., 2016; Tellez et al., 2016). Across settings, small samples and heterogeneous measurement protocols limited comparability and generalizability.

The 17 included studies spanned three broad technological tiers: laboratory‐grade instrumentation, field‐adapted portable systems, and wearable consumer‐grade devices. Laboratory systems predominated in bed rest and simulated confinement protocols and included flywheel resistance devices with force and velocity sensors, short‐arm centrifugation with cardiovascular and neuromuscular monitoring (Kramer et al., 2020, 2021), DXA for assessment of bone and body composition (Smith et al., 2008), ultrasound for vascular and organ morphometry (Arbeille et al., 2024), EEG for assessment of brain electrical activity and neural function (Schneider et al., 2010, 2013), and MRI for structural brain imaging (Roalf et al., 2025).

## 4 DISCUSSION

Structured exercise combined with standardized, context‐aware monitoring might help to mitigate deconditioning and support operational readiness in ICE and analogue missions, particularly with longitudinal behavioural and physiological monitoring (Moraes et al., 2018; Schneider et al., 2013). However, adaptive responses are heterogeneous across ICE settings, and similar outcomes can arise from different combinations of environmental load, confinement stress and exercise exposure (Steinach et al., 2016; Tellez et al., 2016). Figure 2 is a conceptual framework that we propose based on the mapped evidence and supporting physiological literature.

**FIGURE 2:**
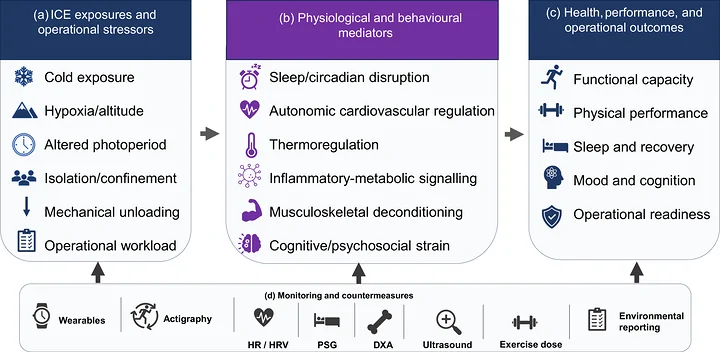
Integrative framework for exercise and physiological monitoring in ICE environments. The framework summarizes our proposed pathway linking ICE‐related environmental and operational stressors to physiological and behavioural mediators and, subsequently, to health, performance and operational outcomes. The bottom panel highlights monitoring tools and countermeasure strategies that might help to characterize exposures, track individual responses and support operational decision‐making. Abbreviations: DXA, dual‐energy X‐ray absorptiometry; HR, heart rate; HRV, heart rate variability; ICE, isolated, confined and extreme; PSG, polysomnography.

### 4.1 Sleep and physical activity in ICE: Interactions modulated by seasonality and confinement

Sleep and daily physical activity patterns in ICE environments appear to be shaped by seasonal photoperiod, confinement and operational routines (Chen et al., 2016; Folgueira et al., 2019), as reported across the Antarctic studies included in this review. Longer sleep duration and delayed awakening during winter, accompanied by reduced spontaneous physical activity, were documented at multiple stations using actigraphy and polysomnography (Collet et al., 2015; Garcia, 2024; Steinach et al., 2016; Tellez et al., 2016). Similar patterns occur in other isolated occupational settings (Miranda et al., 2022), and hypoxia‐related simulations can disrupt sleep further through nocturnal respiratory disturbances (Steinach et al., 2016). Physical activity appears to modulate these effects in part, supporting cognition and buffering psychophysiological stress during confinement (Kraft et al., 2003; Schneider et al., 2013). This protective role of exercise on neurocognitive function was corroborated by Roalf et al. (2025), who found that better sleep quality and consistent training behaviour were associated with attenuated transient grey matter volume changes during Antarctic isolation. Antarctic field exercise data also indicate dynamic cold‐environment responses, with changes in exercise tolerance and submaximal cardiovascular responses across expedition exposure (Moraes et al., 2018). Together, these findings support exercise plus monitoring as an integrated strategy for health and operational readiness in ICE.

A plausible mechanism is that photoperiod disruption and circadian misalignment drive sleep fragmentation, delayed sleep timing and daytime fatigue, with downstream effects on cognition and autonomic regulation. This is consistent with seasonality‐linked sleep/activity shifts in Antarctic isolation, including actigraphy at Comandante Ferraz Station (Collet et al., 2015; Garcia, 2024). The effects of exercise can be context dependent, being beneficial when appropriately timed and dosed but less effective or potentially disruptive during hypoxia or with unfavourable circadian timing (Marchesano et al., 2025; Tellez et al., 2016).

### 4.2 Autonomic and cardiovascular regulation in ICE: HRV as an integrative marker

Autonomic cardiovascular control is a central integrative mechanism linking environmental stressors, sleep disruption and functional performance in ICE and space‐analogue confinement settings (Vigo et al., 2012; Zivi et al., 2020). Among the included studies, heart rate variability (HRV) and cardiovascular autonomic indices were directly assessed in two studies: Moraes et al. (2018) monitored HRV continuously during Antarctic field exercise and documented changes in submaximal cardiovascular responses across the expedition, whereas Kramer et al. (2020) characterized the cardiorespiratory and neuromuscular demand of short‐arm centrifugation in the Artificial Gravity Bed Rest with the European Space Agency (AGBRESA) protocol. HRV‐derived indices are often interpreted as proxies of autonomic balance and recovery status, but they are highly context dependent and sensitive to sleep timing, ambient temperature, physical workload and measurement conditions. Therefore, inconsistent HRV findings across ICE studies might reflect differences in protocol standardization, timing of measurement (day vs. night) and concurrent stressors rather than true physiological disagreement (Moraes et al., 2022; Zivi et al., 2020).

From a mechanistic standpoint, adaptive profiles would be expected to show stabilization or recovery of nocturnal autonomic indices alongside preserved sleep efficiency and functional outcomes. In contrast, maladaptive trajectories would be characterized by progressive deterioration in sleep–autonomic coupling and reduced activity levels, particularly during prolonged confinement or winter periods (Roalf et al., 2025; Steinach et al., 2016). This framing provides a physiologically informed lens to interpret heterogeneous autonomic results and reinforces the need for standardized reporting of measurement context in ICE monitoring protocols.

### 4.3 Cardiovascular remodelling and orthostatic intolerance

In the AGBRESA cohort already included in this review, bed rest reduced left ventricular end‐diastolic volume through a loss of plasma volume, a change that was reversible after resuming ambulation (Hoffmann et al., 2021). Orthostatic tolerance decreased less with daily artificial gravity, regardless of changes in plasma volume (Hoenemann, Moestl, van Herwaarden et al., 2023), while baroreflex‐mediated parasympathetic control was reduced and sympathetic modulation increased during orthostatic stress, which was partly attenuated by artificial gravity (Hoenemann, Moestl, Diedrich et al., 2023). These findings suggest that cardiac remodelling and impaired autonomic baroreflex are distinct mechanisms that overlap only in part, which is consistent with persistent orthostatic intolerance despite preserved cardiorespiratory fitness, as reported by Hedge et al. (2025).

In contrast, vascular compliance itself appeared largely preserved, with aortic compliance, aortic distensibility and pulse wave velocity remaining unchanged after 60 days of bed rest regardless of artificial gravity training, indicating that impaired orthostatic tolerance in this cohort was not driven primarily by large‐artery stiffening (Möstl et al., 2021).

### 4.4 Physiological adaptations: Muscle, bone and cardiorespiratory preservation in confinement and simulated microgravity scenarios

The findings synthesized here indicate that structured exercise protocols, particularly resistance training, flywheel‐based protocols and exercise combined with artificial gravity, can mitigate muscle atrophy, bone mineral loss and cardiorespiratory deconditioning during prolonged unloading protocols. Among bed rest studies, resistance exercise attenuated bone mineral density loss and preserved musculoskeletal function in 60 days of simulated microgravity (Rittweger et al., 2005; Smith et al., 2008), whereas flywheel protocols with adjunct modalities showed partial, although limited, effects on lumbar musculature and spinal health during 90 days of bed rest (Belavý et al., 2011). Daily artificial gravity via short‐arm centrifugation did not preserve aerobic exercise capacity after 60 days of head‐down‐tilt bed rest but mitigated some deterioration in muscle function and neuromuscular performance (Kramer et al., 2021). When aerobic exercise was performed during artificial gravity, cardiorespiratory fitness was preserved in part, although orthostatic tolerance remained impaired (Hedge et al., 2025). Exercise combined with artificial gravity attenuated vascular and organ morphological changes associated with 55 days of bed rest (Arbeille et al., 2024).

These results suggest that no single countermeasure preserves all physiological systems simultaneously; combined or multimodal protocols appear necessary, including resistance exercise for musculoskeletal protection, aerobic training for cardiorespiratory fitness, and artificial gravity or lower‐body negative pressure for orthostatic and haemodynamic stimuli (Evans et al., 2018; Hedge et al., 2022; Linnarsson et al., 2015).

In Antarctic field contexts, physiological outcomes reflect a distinct exposure profile combining cold stress, terrain‐dependent workload and sustained operational activity, which differs substantially from the mechanical unloading that characterizes bed rest protocols (Moraes et al., 2018). Seasonal variation in spontaneous physical activity might additionally influence psychophysiological well‐being across mission phases; specifically, reduced activity during polar winter has been associated with impaired sleep quality and mood disturbance in Antarctic overwintering cohorts (Alvarado et al., 2023; Garcia, 2024; Palinkas & Suedfeld, 2008; Steinach et al., 2016).

### 4.5 Endocrine and metabolic adaptations to unloading

Bed rest combined with resistive vibration exercises, in the Berlin Bed Rest Study, prevented the decrease in sex hormone‐binding globulin observed in inactive control subjects and did not suppress the growth hormone response induced by muscle activity, which would otherwise be attenuated by the rest period (Belavý et al., 2012). In the same cohort, thyroid‐stimulating hormone increased while free triiodothyronine decreased over the course of bed rest, whereas cortisol concentrations remained unchanged throughout the intervention, indicating that unloading altered thyroid axis regulation without evidence of a sustained hypothalamic–pituitary–adrenal stress response (Belavý et al., 2012). At the metabolic level, evidence from sources outside the studies identified in our search indicates that bed rest rapidly reduces insulin sensitivity and GLUT4 translocation to the membrane, along with impaired mitochondrial respiration in skeletal muscle; resistive vibration exercise attenuated the decline in insulin sensitivity during a separate period of bed rest (Kenny et al., 2017).

### 4.6 Immune and inflammatory responses to prolonged unloading

Although not captured by our search strategy, evidence from other bed rest cohorts indicates that the inflammatory signature of prolonged bed rest emerges predominantly during the resumption of physical activity, rather than during the rest period itself, with interleukin‐6, C‐reactive protein and ICAM‐1 showing an increase after the resumption of walking, yet remaining largely stable throughout the bed rest period; exercise during bed rest prevented this post‐rest inflammatory increase (Mutin‐Carnino et al., 2014).

### 4.7 Thermoregulation and environmental physiology in ICE

Thermoregulatory adaptation is another integrative mechanism relevant to ICE physiology, particularly when cold exposure co‐occurs with exercise. Evidence from Antarctic fieldwork indicates that repeated exposure to cold environments combined with sustained physical activity can induce cross‐adaptive responses, including improved heat tolerance during subsequent exercise in thermoneutral conditions, suggesting plasticity in thermoeffector function and central regulatory integration (Moraes et al., 2018). However, environmental conditions, clothing insulation, exercise workload, hydration status and the timing and site of thermal measurements can substantially influence observed responses and limit direct comparisons across studies when reporting is incomplete (Martins et al., 2022; Thuany et al., 2025). Future ICE protocols should standardize environmental and workload reporting and incorporate repeated measures across mission phases to distinguish true adaptation from acute task‐ or context‐dependent variation.

### 4.8 Field biomarker signatures in Antarctic ICE context

Inflammatory and metabolic biomarkers can capture both adaptive and stress‐related responses in ICE. Evidence from Antarctic field camps suggests that short‐term exposure can be accompanied by changes in inflammatory, metabolic, endocrine and microbiome‐related markers, including increased leptin, interleukin‐8 and salivary cortisol, reduced vascular endothelial growth factor, reductions in thyroxine and the Thyroxine/thyroid‐stimulating hormone ratio, and shifts in oral microbiome profiles (Moraes et al., 2023; Thuany et al., 2025). These findings suggest that physiological adaptation in ICE is multisystemic and not restricted to musculoskeletal or cardiorespiratory domains. In practical terms, these biomarker fluctuations are likely to reflect the combined influence of environmental exposure, sleep/circadian disruption, physical workload and energetic/nutritional demands. However, because biomarker outcomes were not the primary focus of most included studies, these findings should be interpreted cautiously. Future studies should combine longitudinal biomarker sampling with standardized timing relative to sleep and exercise and integrate these data with sleep, activity, heart rate and HRV monitoring to distinguish transient adaptive signalling from maladaptive inflammatory load within each operational context (Jordan & Pilla, 2024; Tocci et al., 2024).

### 4.9 Neurovestibular and sensorimotor adaptations

Evidence from spaceflight and bed rest analogues indicates that a reduction in body load during bed rest decreases reliance on proprioceptive stimuli, contributing to deficits in postural stability and locomotor control following bed rest, similar to those observed after spaceflight (Macaulay et al., 2021). In a study lasting 70 days and involving bed rest with the head in a head‐down position, functional mobility and balance performance deteriorated over the course of the intervention, and the exercises (which included resistance and aerobic training) did not prevent this sensorimotor decline, despite their proven benefits for cardiovascular and musculoskeletal outcomes (Koppelmans et al., 2015).

### 4.10 Cognitive and psychosocial effects of stress, fatigue and mood swings on ICE missions

In ICE, cognitive and mental health outcomes are better understood as part of a broader adaptive process shaped by physiological, environmental and social interactions, rather than as isolated consequences of confinement. Structured exercise appears to modulate this process, helping to preserve cognition and reduce mood disturbances during prolonged missions (Kraft et al., 2003; Rittweger et al., 2005; Schneider et al., 2013).

Psychosocial and environmental factors (e.g., social support, spatial perception, habitat characteristics) are likely to influence ICE adaptation and might interact with circadian regularity and structured routines (Marchesano et al., 2025). Emerging technology‐supported operational health frameworks (Urlings et al., 2024) reinforce the value of integrated strategies combining individualized exercise, circadian preservation and supportive psychosocial environments to mitigate stress, fatigue and mood disturbance during prolonged ICE exposure.

### 4.11 Monitoring technologies and methodologies: Advances, gaps and perspectives

Several included studies addressed monitoring strategies in the ICE context and analogous contexts. Antarctic and polar field studies relied on self‐reported records, perceived exertion, actigraphy, autonomic monitoring and minimally invasive tools, reflecting logistical constraints in remote environments (Garcia, 2024; Moraes et al., 2018; Steinach et al., 2016; Tellez et al., 2016). In contrast, confinement and bed rest analogues used more controlled assessments, including neurophysiological, cardiopulmonary, musculoskeletal, vascular and body composition measures (Arbeille et al., 2024; Garcia, 2024; Kraft et al., 2003; Kramer et al., 2020; Moraes et al., 2018; Rittweger et al., 2005; Schneider et al., 2010, 2013; Smith et al., 2008; Steinach et al., 2016; Tellez et al., 2016).

The central challenge is therefore not the availability of technology but the transferability of high‐precision tools to operational ICE settings. Wearables and field‐adapted systems might support longitudinal monitoring, but interpretation requires standardized reporting of timing, posture, ambient conditions and workload (Friedrich et al., 2025; Moraes et al., 2021; Sammito et al., 2024; Urlings et al., 2024).

### 4.12 Limitations

The main limitations across studies reflect small sample sizes typical of extreme and operational settings, frequent restriction to volunteers or military personnel, predominance of male samples, and heterogeneity in exposure characterization, monitoring protocols and physiologically distinct contexts, including Antarctica, bed rest, confinement and artificial gravity. The dependence on subjective measures (self‐reported activity and perceived exertion), limited sensor integration in some contexts, and variable follow‐up windows constrain generalizability and mechanistic interpretation.

The scarcity and heterogeneity of studies precluded quantitative synthesis and limited the ability to standardize effect comparisons across domains. Despite these constraints, this review provides an integrated synthesis of available evidence on physical activity and monitoring strategies in cold‐climate ICE and analogue settings, offering a framework to guide more rigorous, physiologically informed future research.

## 5 CONCLUSION

In conclusion, the evidence synthesized across 17 studies suggests exercise as a pragmatic and domain‐specific countermeasure against deconditioning in ICE and analogue settings, with countermeasure effectiveness varying by exercise modality and target physiological domain. Sleep/circadian disruption and autonomic cardiovascular regulation emerged as integrative pathways linking environmental stressors to performance and health outcomes. However, mechanistic inference remains limited by small samples, heterogeneous exposure profiles and non‐standardized monitoring conditions. The next step is to harmonize monitoring and reporting practices, including sleep and HRV timing, environmental parameters and workload quantification, with longitudinal designs that can disentangle seasonal effects from confinement and exercise. This progression is necessary to enable truly translational, physiologically driven exercise prescriptions and monitoring strategies for polar operations and space‐analogue missions.

## AUTHOR CONTRIBUTIONS

Gustavo S. O. Lima conceived and designed the review, conducted the searches and screening, performed data extraction and synthesis and drafted the manuscript. Leonardo P. Silva contributed to methodological decisions, data synthesis and critical revision. Kaline V. F. Dinis, F. Nayanne B. Sousa, Maria Keiliane F. S. Silva, Danilo C. Talhari and Josélia S. Almeida contributed to data extraction, organization of study materials and manuscript revision. Michele M. Moraes, Rosa M. E. Arantes, Marcos A. Nascimento and Thiago T. Mendes supervised the project, contributed to methodological decisions and interpretation and critically revised the manuscript. All authors approved the final version of the manuscript and agree to be accountable for all aspects of the work in ensuring that questions related to the accuracy or integrity of any part of the work are appropriately investigated and resolved. All persons designated as authors qualify for authorship, and all those who qualify for authorship are listed.

## CONFLICT OF INTEREST

The authors declare no conflicts of interest.

## GENERATIVE AI STATEMENT

Research Rabbit was used as an AI‐assisted citation‐chasing tool to support the identification of potentially relevant studies through reference lists and citation networks. The tool was not used to generate research data, extract data, perform analyses or make scientific interpretations. All records identified through this process were screened and verified by the authors.

## Data Availability

This scoping review is based solely on previously published literature and did not generate primary data. All information supporting the findings is contained within the cited sources.

## Appendix

**TABLE A1:** Technologies used, main outcomes and limitations of the included studies (*n* = 17).

Author/year	Technology	Results	Limitations
(1) Alkner & Tesch (2004)	Rotational decoder with hybrid data sensor for MuscleLab attached to the ergometer	Resistance training mitigated muscle loss and helped maintain physical performance during confinement	Small sample size (4); study in a simulated environment
(2) Schneider et al. (2010)	16‐Channel portable EEG, Brain Vision Analyzer 2.0 software for spectral analysis	Exercise mitigated the physiological and psychological cardiovascular effects of confinement	Small sample (6); simulated environment
(3) Schneider et al. (2013)	16‐Channel portable EEG, Brain Vision Analyzer 2 with sLORETA	Exercise improved prefrontal cortex activity and cognitive performance in prolonged confinement	Small sample (6); simulated environment
(4) Kraft et al. (2003)	Modified perceived exertion scale (activity records) and anthropometric (body weight)	Low perceived load = poorer adaptation; perceived load useful as a marker	Small sample size (8); lack of objective data on volume/intensity; potential influence of motivational factors on exercise load
(5) Roalf et al. (2025)	Structural MRI (FreeSurfer), Bluetooth actigraph, cognition tests (NASA), mood/stress questionnaires	Transient brain changes mostly reversed by 5 months (except thalamus); better sleep/training attenuated effects; cognition remained unclear	Small sample size (25); observational study without experimental control; some crew members without complete data
(6) Rittweger et al. (2005)	Flywheel device (resistance training), cardiovascular and metabolic monitoring	Resistance exercise and pamidronate mitigated bone and muscle loss	Small sample (*n* = 28), microgravity simulation and heterogeneous protocol
(7) Collet et al. (2015)	BodyMedia SenseWear^®^ actigraphy (24 h continuous sleep/activity recording	Sleep was impaired by altitude/seasonality (primarily hypoxia); no group difference in physical activity	Small sample size (26 volunteers); lack of complementary physiological measurements
(8) Tellez et al. (2016)	Complete/portable PSG, Lake Louise AMS score, and exercise diaries (RPE)	Exercise in hypoxia (acute and chronic) exacerbates periodic breathing during sleep	Small sample size (29); self‐reported habitual physical activity
(9) Steinach et al. (2016)	SENSEWEAR Actigraphy (Pro3/MF); bioimpedance/BODPOD	Progressive decline in physical activity level (MET) throughout the winter	Variability between campaigns and seasons
(10) Belavy et al. (2011)	Flywheel; MRI; spinal mobility tests	Exercises had a limited effect on preventing lumbar deconditioning	Small sample (28 volunteers); focus restricted to the lumbar spine
(11) Moraes et al. (2018)	Monark cycle ergometer, gas analysis, HR monitor, temperature sensors, pedometer and portable lactate meter	Increase in total exercise time, reduction in submaximal HR, with no significant change in absolute ${\dot{V}}_{O_{2}\max}$	Only 9 participants (7 civilian researchers + 2 mountaineers); context restricted to Brazilian military personnel
(12) Garcia (2024)	ActTrust (accelerometer/actigraph in wristwatch format)	In winter, sleep increased and wake time delayed; efficiency and sleep quality were preserved despite lower M10AF	Small sample size (*n* = 11); absence of external control group
(13) Smith et al. (2008)	DXA; bone turnover markers; treadmill LBNP; flywheel exercise	Exercise attenuated bone mineral loss during 60 days of simulated microgravity	Small female‐only sample
(14) Kramer et al. (2021)	Short‐arm centrifugation; aerobic and muscle function tests	Artificial gravity did not preserve aerobic capacity but mitigated muscle‐function decline	Small sample; no combined exercise protocol
(15) Kramer et al. (2020)	Short‐arm centrifugation; cardiopulmonary monitoring; EMG	Centrifugation produced cardiovascular and neuromuscular loading	Demand study, not an efficacy trial
(16) Hedge et al. (2025)	Artificial gravity; cycling exercise; cardiorespiratory and orthostatic testing	Exercise during artificial gravity preserved cardiorespiratory fitness but not orthostatic tolerance	Limited transferability beyond bed rest
(17) Arbeille et al. (2024)	Ultrasound; exercise; artificial gravity	Exercise and artificial gravity attenuated vascular, organ and muscle changes	Small sample; ultrasound‐only assessment

Abbreviations: AMS, acute mountain sickness; DXA, dual‐energy X‐ray absorptiometry; HR, heart rate; LBNP, lower‐body negative pressure; M10AF, actigraphy‐derived activity metric during the most active 10 h period; MET, metabolic equivalent; PSG, polysomnography; RPE, subjective perception of exertion; ${\dot{V}}_{O_{2}\max}$, maximum oxygen consumption.

**Data A1**. Complete search strategies, search dates, database‐specific filters and limits, and consolidated results for all databases and additional sources used in this review.

### Search strategy

**1. PubMed/MEDLINE**

2.1 Access and syntax notes

URL: https://pubmed.ncbi.nlm.nih.gov → Advanced Search

MeSH terms are tagged with [MeSH Terms]. Title and abstract fields use [tiab]. Truncation: asterisk (*). Boolean operators in uppercase. Human filter: “humans” [MeSH Terms].

**1.1 Search String (May 2026)**

(Antarctic* OR “polar station” OR “polar expedition” OR “polar mission” OR “polar environment” OR overwintering OR “winter‐over” OR “extreme environment” OR “harsh environment” OR “remote environment” OR “Space Flight” OR spaceflight OR “Space Simulation” OR “space mission” OR “space analogue” OR “space analog” OR “Mars simulation” OR Mars500 OR “MARS‐500” OR HERA OR “HI‐SEAS” OR “lunar analogue” OR “long‐duration mission” OR “long duration spaceflight” OR microgravity OR “simulated microgravity” OR “microgravity analogue” OR “microgravity analog” OR weightlessness OR “bed rest” OR bedrest OR “head‐down tilt” OR “head‐down bed rest” OR “anti‐orthostatic” OR “dry immersion” OR “hypobaric chamber” OR “hypoxic chamber” OR “high altitude” OR submarine OR “undersea habitat”)

AND

(isolation OR isolated OR confinement OR confined OR “prolonged confinement” OR “long‐term confinement” OR “social isolation” OR “hermetic isolation” OR “hermetically sealed” OR quarantine OR “crew isolation” OR “expedition member” OR overwintering OR “winter‐over” OR “ICE environment” OR “ICE condition” OR “isolated confined extreme”)

AND

(exercise OR “physical exercise” OR “exercise training” OR “exercise intervention” OR “physical activity” OR “spontaneous physical activity” OR “habitual physical activity” OR “daily physical activity” OR “resistance training” OR “strength training” OR “resistance exercise” OR “flywheel exercise” OR “flywheel training” OR “gravity‐independent exercise” OR “aerobic training” OR “aerobic exercise” OR “endurance training” OR “combined training” OR countermeasure* OR “exercise countermeasure” OR “physical fitness” OR “physical performance” OR “functional capacity” OR VO2max OR “maximal oxygen uptake” OR “cardiorespiratory fitness” OR “physiological monitoring” OR wearable* OR actigraphy OR accelerometr* OR “heart rate variability” OR HRV OR “sleep monitoring” OR polysomnography OR deconditioning OR “physical deconditioning” OR “muscle atrophy” OR “bone loss” OR “bone density”)

AND humans

**1.2 Results log PubMed**

**Table:**

Search	Date	Records retrieved
Search	May 2026	272

**2. WEB OF SCIENCE**

**2.1 Access and syntax notes**

URL: https://www.webofscience.com → Advanced Search

All terms use TS = (Topic Search), which covers title, abstract, keywords and Keywords Plus. No MeSH tags. Truncation: asterisk (*). Boolean operators in uppercase. Document type filters applied after search execution.

**2.2 Search string (May 2026)**

TS =(Antarctic* OR “polar station” OR “polar expedition” OR “polar mission”OR “polar environment” OR overwintering OR “winter‐over” OR “extreme environment” OR “harsh environment” OR isolation OR confinement OR “remote environment” OR spaceflight OR “space flight” OR “space mission” OR “space simulation” OR “space analogue” OR “space analog”OR “Mars simulation” OR Mars500 OR “MARS‐500” OR HERA OR “HI‐SEAS” OR “lunar analogue” OR “long‐duration mission” OR “long duration spaceflight” OR microgravity OR “simulated microgravity” OR “microgravity analogue” OR “microgravity analog” OR weightlessness OR “bed rest” OR bedrest OR “head‐down tilt” OR “head‐down bed rest” OR “anti‐orthostatic” OR “dry immersion” OR “hypobaric chamber” OR “hypoxic chamber” OR “high altitude” OR submarine OR “undersea habitat”)

AND

TS =(exercise OR “physical exercise” OR “exercise training” OR “exercise intervention” OR “physical activity” OR “spontaneous physical activity” OR “habitual physical activity” OR “daily physical activity” OR “resistance training” OR “strength training” OR “resistance exercise” OR “flywheel exercise” OR “flywheel training” OR “gravity‐independent exercise” OR “aerobic training” OR “aerobic exercise” OR “endurance training” OR “combined training” OR countermeasure* OR “exercise countermeasure” OR “physical fitness” OR “physical performance” OR “functional capacity” OR VO2max OR “maximal oxygen uptake” OR “cardiorespiratory fitness” OR “physiological monitoring” OR wearable* OR actigraphy OR accelerometr* OR “heart rate variability” OR HRV OR “sleep monitoring” OR polysomnography OR deconditioning OR “physical deconditioning” OR “muscle atrophy” OR “bone loss” OR “bone density”)

**2.3 Results log WEB OF SCIENCE**

**Table:**

Search	Date	Records retrieved
Search	May 2026	256

**3. SCOPUS**

**3.1 Access and syntax notes**

URL: https://www.scopus.com → Advanced Search

All terms use TITLE‐ABS‐KEY(…), which covers title, abstract and author keywords. No controlled vocabulary equivalent to MeSH. Truncation: asterisk (*). Boolean operators in uppercase. Document type and subject area filters applied after search execution.

**3.2 Search string (May 2026)**

(Antarctic* OR “polar station” OR “polar expedition” OR “polar mission” OR “polar environment” OR overwintering OR “winter‐over” OR “extreme environment” OR “harsh environment” OR “remote environment” OR spaceflight OR “space flight” OR “space mission” OR “space simulation” OR “space analogue” OR “space analog” OR “Mars simulation” OR Mars500 OR “MARS‐500” OR HERA OR “HI‐SEAS” OR “lunar analogue” OR “long‐duration mission” OR “long duration spaceflight” OR microgravity OR “simulated microgravity” OR “microgravity analogue” OR “microgravity analog” OR weightlessness OR “bed rest” OR bedrest OR “head‐down tilt” OR “head‐down bed rest” OR “anti‐orthostatic” OR “dry immersion” OR “hypobaric chamber” OR “hypoxic chamber” OR “high altitude” OR submarine OR “undersea habitat”)

AND

(isolation OR isolated OR confinement OR confined OR “prolonged confinement” OR “long‐term confinement” OR “social isolation” OR “hermetic isolation” OR “hermetically sealed” OR quarantine OR “crew isolation” OR “expedition member” OR overwintering OR “winter‐over” OR “ICE environment” OR “ICE condition” OR “isolated confined extreme”)

AND

(exercise OR “physical exercise” OR “exercise training” OR “exercise intervention” OR “physical activity” OR “spontaneous physical activity” OR “habitual physical activity” OR “daily physical activity” OR “resistance training” OR “strength training” OR “resistance exercise” OR “flywheel exercise” OR “flywheel training” OR “gravity‐independent exercise” OR “aerobic training” OR “aerobic exercise” OR “endurance training” OR “combined training” OR countermeasure* OR “exercise countermeasure” OR “physical fitness” OR “physical performance” OR “functional capacity” OR VO2max OR “maximal oxygen uptake” OR “cardiorespiratory fitness” OR “physiological monitoring” OR wearable* OR actigraphy OR accelerometr* OR “heart rate variability” OR HRV OR “sleep monitoring” OR polysomnography OR deconditioning OR “physical deconditioning” OR “muscle atrophy” OR “bone loss” OR “bone density”)

AND

DOCTYPE (ar OR re) AND SUBJAREA (Medi OR Heal OR Mult OR Bioc OR Neur OR Envi OR eart).

**3.3 Results log SCOPUS**

**Table:**

Search	Date	Records retrieved
Search	May 2026	485

**4. EMBASE**

4.1 Access and syntax notes

URL: https://www.embase.com → Advanced Search

EMBASE uses Emtree, its own controlled vocabulary. Emtree terms are followed by ‘/exp’ to include all narrower terms (exploded). Free‐text terms in title and abstract use the suffix:ti, ab. Truncation: asterisk (*). Boolean operators in uppercase. Limit to human studies and article/review document types as shown.

**4.2 Search string (May 2026)**

(‘space flight’/exp OR ‘weightlessness’/exp OR ‘bed rest’/exp OR antarctic*:ti, ab OR ‘polar station’:ti, ab OR ‘polar expedition’:ti, abOR ‘polar mission’:ti, ab OR ‘polar environment’:ti, ab OR overwintering:ti, ab OR ‘winter‐over’:ti, ab OR ‘isolated confined extreme’:ti, ab OR ‘ice environment’:ti, ab OR spaceflight:ti, ab OR ‘space flight’:ti, ab OR ‘space mission’:ti, ab OR ‘space simulation’:ti, ab OR ‘space analogue’:ti, ab OR ‘space analog’:ti, ab OR ‘mars simulation’:ti, ab OR mars500:ti, ab OR ‘mars‐500’:ti, ab OR hera:ti, ab OR ‘hi‐seas’:ti, ab OR ‘lunar analogue’:ti, ab OR ‘long‐duration mission’:ti, ab OR ‘long duration spaceflight’:ti, ab OR microgravity:ti, ab OR ‘simulated microgravity’:ti, ab OR ‘microgravity analogue’:ti, ab OR ‘microgravity analog’:ti, ab OR ‘head‐down bed rest’:ti, ab OR ‘dry immersion’:ti, ab OR ‘undersea habitat’:ti, ab OR submarine:ti, ab)

AND

(isolation:ti, ab OR isolated:ti, ab OR confinement:ti, ab OR confined:ti, ab OR ‘prolonged confinement’:ti, ab OR ‘long‐term confinement’:ti, ab OR ‘crew isolation’:ti, ab OR ‘expedition member’:ti, ab OR overwintering:ti, ab OR ‘winter‐over’:ti, ab OR ‘isolated confined extreme’:ti, ab OR ‘ice environment’:ti, ab)

AND

(‘exercise’/exp OR ‘physical activity’/exp OR ‘strength training’/exp OR exercise:ti, ab OR ‘physical exercise’:ti, ab OR ‘exercise training’:ti, ab OR ‘exercise intervention’:ti, ab OR ‘physical activity’:ti, ab OR ‘resistance training’:ti, ab OR ‘strength training’:ti, ab OR ‘aerobic exercise’:ti, ab OR ‘endurance training’:ti, ab OR countermeasure*:ti, ab OR ‘exercise countermeasure’:ti, ab OR ‘physical fitness’:ti, ab OR ‘physical performance’:ti, ab OR ‘functional capacity’:ti, ab OR vo2max:ti, ab OR ‘maximal oxygen uptake’:ti, ab OR ‘cardiorespiratory fitness’:ti, ab OR ‘physiological monitoring’:ti, ab OR wearable*:ti, ab OR actigraphy:ti, ab OR accelerometr*:ti, ab OR ‘heart rate variability’:ti, ab OR hrv:ti, ab OR deconditioning:ti, ab OR ‘physical deconditioning’:ti, ab)

AND [humans]/limAND [article]/lim

**4.3 Results log EMBASE**

**Table:**

Search	Date	Records retrieved
Search	May 2026	208

**5. Consolidated results across all databases**

**Table:**

Database	Search
PubMed/MEDLINE	272
Web of Science	256
Scopus	485
EMBASE	208
Grey literature (BDTD)	246
Citation chasing (research rabbit)	11
**Total**	**1478**
